# Polystyrene Microplastics Exacerbate Systemic Inflammation in High-Fat Diet-Induced Obesity

**DOI:** 10.3390/ijms241512421

**Published:** 2023-08-04

**Authors:** Aden Geonhee Lee, Sora Kang, Hye Ji Yoon, Suyeol Im, Seung Jun Oh, Youngmi Kim Pak

**Affiliations:** 1 Phillips Exeter Academy, Exeter, NH 03833, USA; adenlkorea@gmail.com; 2 Department of Neuroscience, Graduate School, Kyung Hee University, Seoul 02447, Republic of Korea; ksr8947@khu.ac.kr (S.K.); yhj2271@khu.ac.kr (H.J.Y.); 3 Department of Biomedical Sciences, Graduate School, Kyung Hee University, Seoul 02447,Republic of Korea; suryeol@khu.ac.kr (S.I.); ohsungjun124@naver.com (S.J.O.); 4 Department of Physiology, School of Medicine, Biomedical Science Institute, Kyung Hee University, Seoul 02447, Republic of Korea

**Keywords:** polystyrene, microplastics, BV2 cells, high-fat diet, obesity, inflammation

## Abstract

Microplastics (MPs) are recognized as environmental pollutants with potential implications for human health. Considering the rapid increase in obesity rates despite stable caloric intake, there is a growing concern about the link between obesity and exposure to environmental pollutants, including MPs. In this study, we conducted a comprehensive investigation utilizing in silico, in vitro, and in vivo approaches to explore the brain distribution and physiological effects of MPs. Molecular docking simulations were performed to assess the binding affinity of three plastic polymers (ethylene, propylene, and styrene) to immune cells (macrophages, CD4^+^, and CD8^+^ lymphocytes). The results revealed that styrene exhibited the highest binding affinity for macrophages. Furthermore, in vitro experiments employing fluorescence-labeled PS-MPs (fPS-MPs) of 1 μm at various concentrations demonstrated a dose-dependent binding of fPS-MPs to BV2 murine microglial cells. Subsequent oral administration of fPS-MPs to high-fat diet-induced obese mice led to the co-existence of fPS-MPs with immune cells in the blood, exacerbating impaired glucose metabolism and insulin resistance and promoting systemic inflammation. Additionally, fPS-MPs were detected throughout the brain, with increased activation of microglia in the hypothalamus. These findings suggest that PS-MPs significantly contribute to the exacerbation of systemic inflammation in high-fat diet-induced obesity by activating peripheral and central inflammatory immune cells.

## 1. Introduction

Plastics are inexpensive, lightweight, and resistant to water and decay. They are frequently used in daily life, both at home and in industry [1]. The benefits of plastics make it virtually impossible to completely ban their use. However, plastics, especially secondary microplastics (MPs) [2] generated by abrasion, degradation, corrosion, photo-oxidation, and biological transformation of larger plastics, pollute the environment and pose risks to the environment, wildlife, and humans [3,4]. There are several reports of MPs being found in food, particularly in seafood, sea salt, and drinking water [5]. MPs have also been found in the gastrointestinal tract of marine animals, the human intestine and placenta, and other tissues. Ingested MPs are inert foreign bodies to the host organism, but they have shown harmful effects by releasing other chemicals, such as environmental pollutants and plastic additives, and triggering local immune responses.

MPs are characterized as plastic particles less than 5 mm in size. MPs larger than 50 μm are not absorbed, while microplastics smaller than 1 μm (nanoplastics) do not significantly accumulate [6]. Therefore, the concern for human health lies in the effects of MPs between 1 and 50 μm. The most common polymer types of secondary MPs used in practice are polyethylene (PE), polypropylene (PP), and polystyrene (PS) [7,8]. The major sources of MP exposure are thought to be ingestion of drinking water in plastic bottles and inhalation of indoor air. It is estimated that humans ingest tens of thousands to millions of these MP particles per year, or a few milligrams per day [4]. Once ingested, >90% of large MPs (>150 μm) are reported to be excreted in feces, but smaller particles can cross the blood–brain barrier (BBB), placenta, and epithelium, depending on particle size. MPs <2.5 μm can enter the systemic circulation by endocytosis [9,10]. Although plastics were thought to be inert, there have been several reports that exposure to MPs can alter energy and lipid metabolism through the activation of obesogenic mechanisms.

Obesity rates have continuously increased over the last decades, even though caloric intake and energy expenditure have remained similar [11]. Thus, a high-calorie diet alone cannot explain the increase in obesity in recent years, raising the possibility that other environmental factors may play a role in obesity, including increased exposure to MPs [4]. High-fat diet (HFD) induces a chronic and systemic low-grade inflammation state and induces proinflammatory cytokines such as TNF-α, IL-6, and IL-1β and chemokines such as MCP-1 in various tissues [12]. Inflammatory cytokines disrupt the blood–brain barrier (BBB), promoting chemokine-recruited monocytes to cross the BBB and transform into inflammatory microglia [13]. Newly activated microglia induce neuroinflammation, increase oxidative stress, and decrease mitochondrial function, leading to neuronal cell death by apoptosis or necrosis. Therefore, HFD-induced chronic inflammation may facilitate the uptake of MPs into the organism and aggravate the deleterious effects of MPs as obesogens. However, the effects of the ingested MPs on the endocrine, nervous, and immune systems in HFD-induced obesity are not well understood.

In this study, we performed in silico, in vitro, and in vivo studies to investigate what types of MP polymers affect human immune cells and whether MP aggravates HFD-induced obesity-related parameters and systemic inflammation.

## 2. Results

### 2.1. In Silico Study

#### Molecular Docking

**Figure 1:**
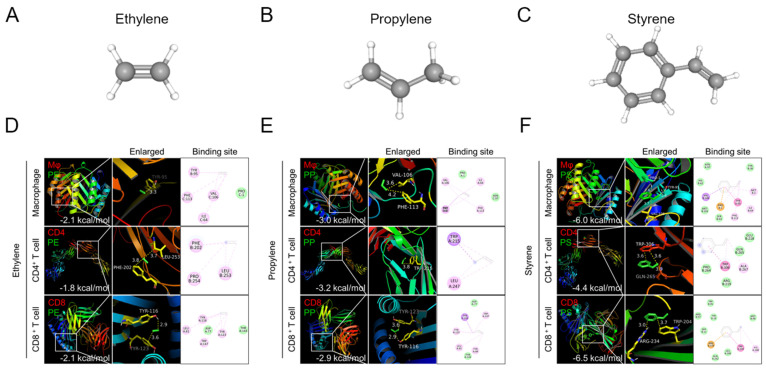
Molecular docking binding affinity of plastic polymers for macrophages, CD4^+^ T cells, and CD8^+^ T cells. Structures of (**A**) ethylene, (**B**) propylene, and (**C**) styrene. (**D**–**F**) Three-dimensional structures of migration inhibitory factor (MIF) for macrophages, CD4 for CD4^+^ T cells, and CD8 for CD8^+^ T cells were obtained from the PDB database. The white boxes of the polymer binding sites are enlarged in the second panels. The binding affinity scores are shown at the bottom of the first panels.

To investigate which types of MP polymers can bind to immune cells, we performed molecular docking analysis between three major types of plastic polymers (ethylene, propylene, and styrene) (Figure 1A–C) and three human immune cells (macrophages and CD4^+^ and CD8^+^ T cells). Three-dimensional crystal structures of representative surface proteins of the three immune cells were obtained from the PDB database. These were the migration inhibitory factor (MIF, PDB ID: 1GD0) for macrophages, the T-cell surface glycoprotein CD4 (PDB ID: 1WIP) for CD4^+^ T cells, and the T-cell coreceptor CD8 (PDB ID: 1AKJ) for CD8^+^ T cells.

Molecular docking simulation showed that styrene has the highest binding affinity scores with CD4 (−4.4 kcal/mol), CD8 (−6.5 kcal/mol), and MIF (−6.0 kcal/mol) (Figure 1D–F). But ethylene showed the lowest binding affinity with MIF, CD4, and CD8 (Figure 1D). Propylene also has a relatively low binding affinity (Figure 1E). Since a more negative binding affinity score means better binding of the plastic polymer to immune cells, styrene is most likely to bind to immune cells, while ethylene and propylene are less likely to bind to immune cells. Therefore, we selected styrene among three plastic polymers and conducted the following in vitro and in vivo experiments.

### 2.2. In Vitro Study

#### Polystyrene Microplastics Bind to Microglia in BV2 Cells

**Figure 2:**
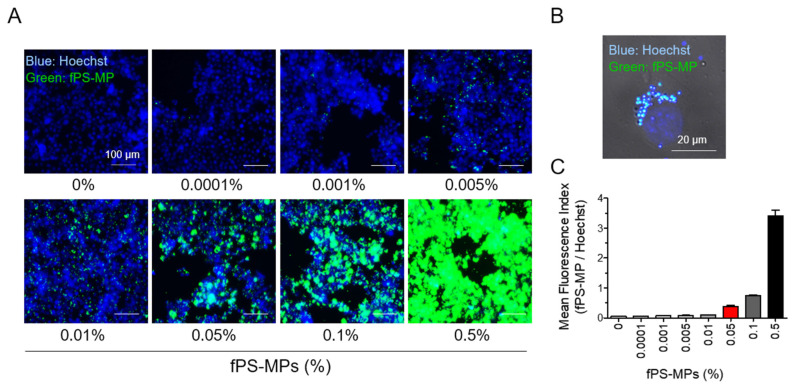
Uptake of fPS-MPs by BV2 microglial cells. BV2 cells were incubated with different concentration of fPS-MPs (0–0.5%) for 24 h and visualized under a fluorescence microscope. (**A**) Representative images taken with the iRiS Digital Cell Imaging System. fPS-MPs (green) are bound to microglial cells (nuclei, blue, Hoechst). Scale bar: 100 µm. (**B**) Confocal image of 0.05% fPS-MP-treated microglial cells. Differential interface contrast (DIC) images were overlaid with green (fPS-MP) and blue (Hoechst) fluorescence images. Scale bar: 20 µm. (**C**) Dose-dependent graph of mean fluorescence index (fPS-MP/Hoechst).

An in vitro experiment was performed using fluorescence-labeled PS-MPs (fPS-MPs) to investigate whether polystyrene-microplastics (PS-MPs) can bind microglia cells. After BV2 cells were incubated with various concentrations of fPS-MPs (0~0.5%) for 24 h, fluorescent particles in the cells were analyzed. The number of fluorescent particles in fPS-MPs was 4,750,000 in 0.1% fPS-MPs and 2,375,000 in 0.05% fPS-MPs. Fluorescent particles were present in and/or on the cells in all concentrations (Figure 2A).

The enlarged confocal image demonstrates that fPS-MP particles were indeed present inside the BV2 cells (Figure 2B). The mean fluorescence index (fPS-MP fluorescence/Hoechst) increased linearly with fPS-MP concentration (Figure 2C). These results suggest that, as predicted by molecular docking analysis, most fPS-MPs can bind to microglia in a concentration-dependent manner and be taken up by phagocytosis (possibly macropinocytosis). Based on the results of the fluorescence index (Figure 2C) and previous studies [4,5], 0.05% fPS-MPs was considered an appropriate concentration for in vivo administration.

### 2.3. In Vivo Study

#### 2.3.1. Effects of fPS-MPs on Metabolic Profiles

**Figure 3:**
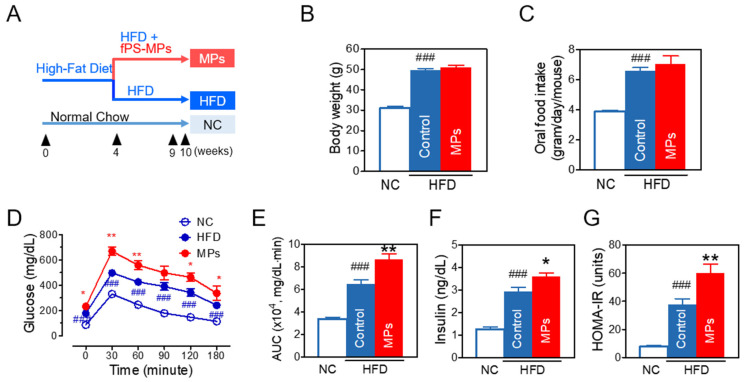

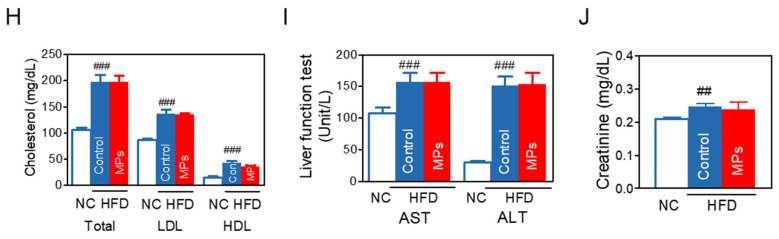
Effects of fPS-MPs on metabolic parameters in HFD-induced obese mice. (**A**) Experimental scheme in vivo; (**B**) body weight at the end of the experiment; (**C**) oral food intake; (**D**) time-course changes of blood glucose in the oral glucose tolerance test (OGTT) at 9 weeks; (**E**) area under the curve (AUC) of the OGTT; (**F**) fasting insulin; (**G**) homeostatic model assessment of insulin resistance (HOMA-IR); (**H**) cholesterol (Total-C, LDL-C, HDL-C); (**I**) liver function enzymes of AST and ALT; (**J**) renal function marker of creatinine. Data are expressed as the mean ± SEM (*n* = 5). ^##^ *p* < 0.01 and ^###^ *p* < 0.001 vs. NC, and * *p* < 0.05 and ** *p* < 0.01 vs. HFD-control. NC, normal chow; Control, HFD-control; MPs, HFD plus fPS-MPs 0.125 µg/mouse per day.

To study the effect and distribution of PS-MPs, mice were fed a HFD for 4 weeks, and subsequently fPS-MPs (0.125 µg/mouse, 2.375 × 10^6^ MPs/mouse per day) were orally administered for 6 weeks (Figure 3A for scheme). As expected, mice fed a HFD for 10 weeks (HFD group) had increased body weight (Figure 3B), food intake (Figure 3C), and calorie intake. However, oral administration of fPS-MPs for 6 weeks (MPs group) did not further increase body weight or food intake.

The HFD group showed a significant increase in blood glucose and the area under the curve (AUC) of glucose in an oral glucose tolerance test (OGTT) (Figure 3D,E), fasting insulin (Figure 3F), and HOMA-IR (Figure 3G) compared to the normal chow (NC) group. Interestingly, the MPs group further significantly increased blood glucose levels, the AUC of glucose in an OGTT (Figure 3D,E), and fasting insulin levels (Figure 3F), and worsened insulin resistance in comparison to the HFD group (Figure 3G) (*p* < 0.01).

Similarly to body weight, HFD increased lipid levels of total cholesterol, LDL-cholesterol, and HDL-cholesterol (Figure 3H) and damaged hepatic (Figure 3I) and renal function (Figure 3J), while fPS-MPs did not worsen lipid profiles or hepatic or renal function.

#### 2.3.2. Effects of fPS-MPs on Abnormal Fat Accumulation in Adipose Tissue and Liver

**Figure 4:**
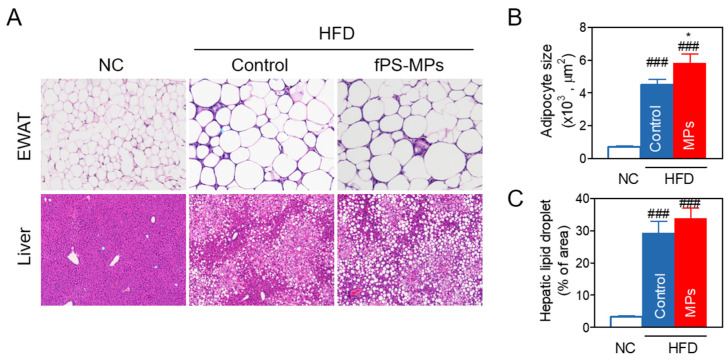
Fat accumulation of the epididymal white adipose tissue (EWAT) and liver. (**A**) H&E staining of EWAT and the liver; (**B**) adipocyte size of EWAT; (**C**) % of area of liver lipid droplet. ^###^ *p* < 0.001 vs. NC, and * *p* < 0.05 vs. HFD-control. NC, normal chow; Control, HFD-control; MPs, HFD plus fPS-MPs 0.125 µg/mouse per day.

Since fat accumulation in the epididymal fat and liver causes insulin resistance and increases circulating inflammatory immune cells and cytokines [14], we histologically evaluated fat accumulation. Fat accumulation in epididymal white adipose tissue (EWAT) or liver was measured by the epididymal adipocyte size and the % of area of hepatic lipid droplets (Figure 4A–C). fPS-MPs further increased adipocyte size (Figure 4B, *p* < 0.05) compared to the HFD group. H&E staining showed the exacerbation of hepatic steatosis (Figure 4A), but the % of area of hepatic lipid droplets was not significantly changed (Figure 4C).

#### 2.3.3. Blood Immune Cells Binding to PS-MPs

**Figure 5:**
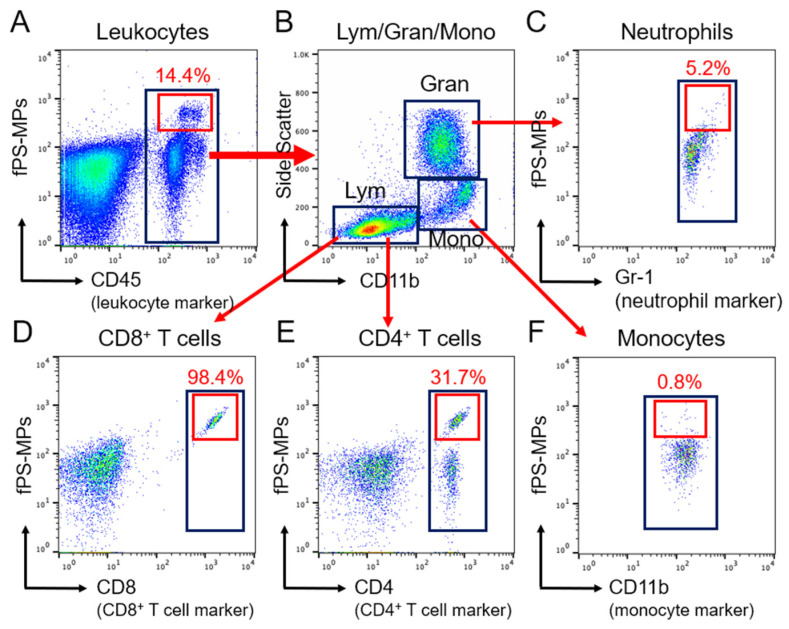
Flow cytometric gating strategy for blood immune cells bound to fPS-MP microplastics. (**A**) Total CD45^+^ leukocytes (black box) and fPS-MPs binding leukocytes (red box). (**B**) Separation of the population of CD45^+^ leukocytes in the black box of panel A into lymphocytes (Lym), granulocytes (Gran), and CD11b^+^ monocytes (Mono) (black boxes). (**C**–**F**) Each population was separated from B using the following markers: (**C**) Gr-1^+^ neutrophils (black box); (**D**) CD8^+^ T cells (black box); (**E**) CD4^+^ T cells (black box); (**F**) CD11b^+^ monocytes (black box). In (**A**–**F**), the red boxes indicate co-labeling for fPS-MP microplastic and marker-specific positive cells.

To investigate which blood leukocytes bind to polystyrene microplastics, we analyzed blood immune cells, including monocytes, neutrophils, and CD4^+^ and CD8^+^ lymphocytes. In the blood immune cell analysis, over 99% of fPS-MPs were captured by leukocytes, which make up 14.4% of the total leukocytes (Figure 5A). In particular, 98.4% of CD8^+^ T cells and 31.7% of CD4^+^ T cells were bound to fPS-MPs (Figure 5B,D,E). In comparison, the proportion of neutrophils bound to fPS-MPs was 5.2% (Figure 5C), and that of monocytes was 0.8% (Figure 5F). These results suggest that fPS-MPs can circulate in the blood while being captured by leukocytes and induce an immune response.

#### 2.3.4. Impact of fPS-MPs on Immune Cells in Obese Mice

**Figure 6:**
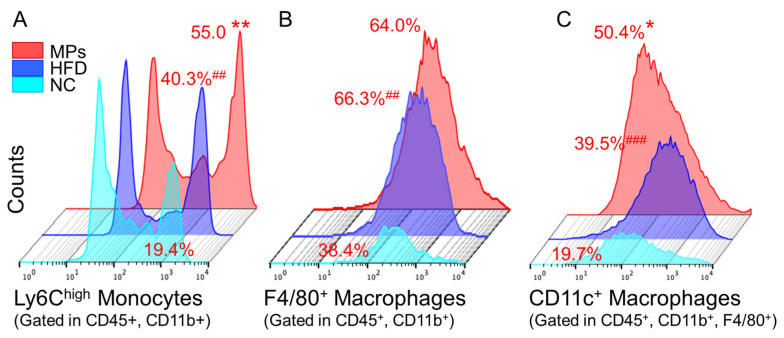
Inflammatory immune cells in the blood and adipose tissue. (**A**) Inflammatory Ly6C^high^ monocytes in the blood; (**B**) F4/80^+^ macrophages in the adipose tissue; (**C**) CD11c^+^ inflammatory macrophages in the adipose tissue. ^##^ *p* < 0.01 and ^###^ *p* < 0.001 vs. NC, and * *p* < 0.05 and ** *p* < 0.01 vs. HFD-control. NC, normal chow; Control, HFD-control; MPs, HFD plus fPS-MPs 0.125 µg/mouse per day.

In response to pro-inflammatory stimuli, Ly6C^high^ monocytes, precursors of inflammatory macrophages, differentiate into CD11c^+^ M1-type macrophages in various organs and promote inflammation [12,15]. Therefore, we investigated whether fPS-MPs can be an inflammatory stimulus for the activation of Ly6C^high^ inflammatory monocytes and CD11c^+^ macrophages. Flow cytometric analysis of immune cells revealed that fPS-MPs further increased pro-inflammatory Ly6C^high^ monocytes in the blood (Figure 6A) and proinflammatory CD11c^+^ macrophages (Figure 6C) in the EWAT compared to HFD alone. However, there were no changes in F4/80^+^ macrophages in the adipose tissue (Figure 6B) or CD4^+^ T cells or CD8^+^ T cells in the blood by fPS-MPs. These results suggest that fPS-MPs exacerbate peripheral inflammation, glucose metabolism, and insulin resistance by increasing fat accumulation in adipose tissue and recruiting blood-inflammatory Ly6C^high^ monocytes in adipose tissue and converting them into CD11c^+^ macrophages.

#### 2.3.5. Deposition of fPS-MPs in the Brain

**Figure 7:**
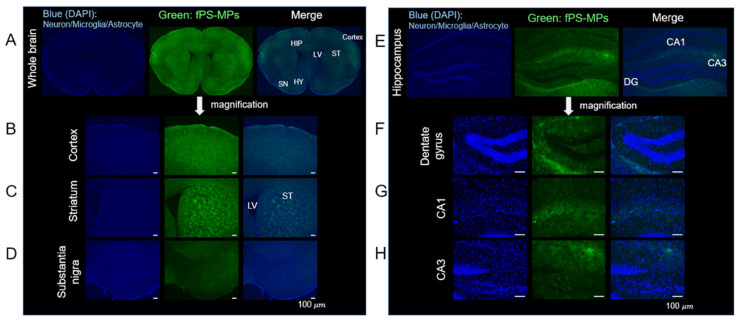
fPS-MP deposition in the brain. Green, fluorescent particles were detected in most regions of the brain. Representative images taken with the iRiS Digital Cell Imaging System are shown. Brain coronal sections from HFD-induced obese mice treated orally with fPS-MPs (0.125 µg/mouse per day) for 6 weeks show fPS-MPs (green) bound to cells (nuclei, blue, Hoechst). Scale bar: 100 µm. Regions marked: (**A**) whole brain (HY, hypothalamus; LV, lateral ventricle); (**B**) cortex; (**C**) striatum (ST); (**D**) substantia nigra (SN); (**E**) hippocampus (HIP); (**F**) dentate gyrus (DG); (**G**) hippocampal CA1; and (**H**) hippocampal CA3.

Although it has been reported that nanoplastics can cross the BBB and accumulate in the brain [16], there have been few in vivo studies of PS-MPs. After oral administration of fPS-MPs to HFD-induced obese mice for 6 weeks, fPS-MPs were detected in most regions of the brain (Figure 7A). This included the cortex (Figure 7B), striatum (Figure 7C), substantia nigra (Figure 7D), and hippocampus (Figure 7E–H), with the most pronounced deposition of fPS-MPs in the cortex, striatum, and hippocampus.

#### 2.3.6. Effects of MPs on Neuroinflammation in the Hypothalamus of the Brain

**Figure 8:**
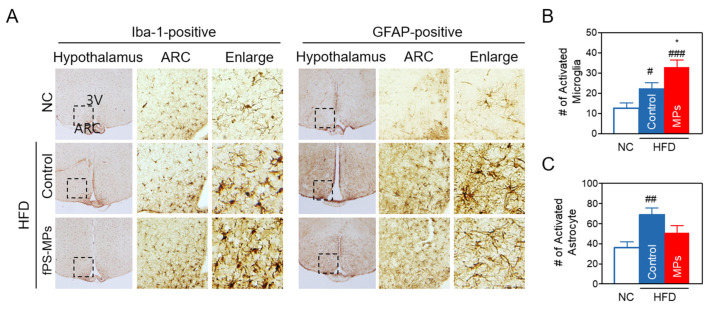
Polystyrene microplastics exacerbate HFD-induced hypothalamic inflammation. (**A**) Representative images and higher magnification inset of the hypothalamus of mice. Hypothalamic sections were immunostained with GFAP or Iba-1 antibodies. The arcuate nucleus (ARC) area (arrow, inset box) and the third ventricle (3V) are indicated. The box area in ARC was enlarged to view the cellular morphology (Enlarge). (**B**) Number of GFAP-positive activated astrocytes and (**C**) Iba-1-positive activated microglia in the ARC. Data are expressed as the mean ± SEM (*n =* 5). ^#^ *p* < 0.05, ^##^ *p* < 0.01, and ^###^ *p* < 0.001 vs. NC, and * *p* < 0.05 vs. HFD-control. NC, normal chow; Control, HFD-control; MPs, HFD plus fPS-MPs 0.125 µg/mouse per day.

HFD-induced obesity increases neuroinflammation in the hippocampus and hypothalamus by allowing inflammatory immune cells to cross the BBB [13]. We investigated whether fPS-MPs could exacerbate hypothalamic inflammation in HFD-induced obese mice. Immunohistochemical staining of brain coronal sections for microglia and astrocytes revealed that HFD increased the accumulation of activated GFAP-positive astrocytes and Iba-1-positive microglia in the hypothalamus (Figure 8A–C), in addition to exhibiting distinct morphological changes with hypertrophied and enlarged soma size with an amoeboid shape (Figure 8A).

The fPS-MPs (MPs group) increased the number of activated Iba1-positive microglia with worse morphological changes than the HFD group (Figure 8A,B). However, the number of activated GFAP-positive astrocytes was rather decreased compared to the HFD group (Figure 8C). Considering that fPS-MPs bind to BV2 microglial cells and increase blood Ly6C^high^ inflammatory monocytes and CD11c^+^ recruited macrophages in adipose tissue, it is possible that fPS-MPs may activate the resident microglial cells, and the increased Iba1-positive activated microglia may be derived from blood Ly6C^high^ inflammatory monocytes, similarly to adipose tissue macrophages.

## 3. Discussion

This study reports the first finding that PS-MPs can penetrate the BBB and exacerbate brain neuroinflammation in obese patients. Fat accumulation due to overnutrition leads to hypoxic cell damage, which stimulates the infiltration of Ly6C^high^ inflammatory monocytes into various tissues, where they are more likely to differentiate into inflammatory macrophages [17]. The newly recruited inflammatory macrophages release multiple inflammatory cytokines, leading to peripheral inflammation and insulin resistance in adipose tissue, the liver, and skeletal muscle. In addition, the circulating Ly6C^high^ inflammatory monocytes cross the BBB and differentiate into microglia at the arcuate nucleus (ARC) of the hypothalamus [18]. Thus, the activation and increase of these microglia can lead to a vicious cycle of macrophage recruitment and inflammatory cytokine production, resulting in chronic peripheral and central inflammation. Interestingly, our study demonstrates that PS-MPs exhibit a high affinity for binding to CD4^+^ and CD8^+^ T cells in the blood; however, this binding does not result in an increase in the number of these T cell subsets. Conversely, PS-MPs display lower binding to monocytes, but significantly enhance the population of inflammatory Ly6C^high^ monocytes. These results suggest that PS-MPs-induced inflammation may be potentially mediated by circulating blood monocytes rather than CD4^+^ and CD8^+^ T cells.

Microglia and astrocytes are the major glial cells in the mammalian brain. Under normal conditions, microglia are not replenished by peripheral immune cells and are maintained by slow self-renewal. Microglia naturally remove neuron debris, while astrocytes control neuronal homeostasis, synaptic transmission, and plasticity [19]. The characteristic long processes of astrocytes surround blood vessels, maintain BBB integrity, and regulate microglial activity; however, obesity alters their plasticity and function and converts microglia into an activated form to clear synaptic debris in the hypothalamus [20]. Reactive astrocytes also activate microglia and contribute to the aggravation of hypothalamic inflammation. We found that PS-MPs increased the number of activated microglia in the hypothalamus, but not the number of reactive astrocytes. Because the molecular crosstalk between microglia and astrocytes is complicated in the obese state and in hypothalamic inflammation, it is difficult to interpret the reduction of reactive astrocytes by fPS-MPs as beneficial. Depending on the context, reactive astrocytes can adopt multiple states, gaining some protective or detrimental functions that can occur simultaneously. Combined with in vitro and in vivo results, PS-MPs may directly activate microglia and indirectly recruit peripheral macrophages. Therefore, it is thought that the increase in activated microglia derived from peripheral monocytes, rather than microglia present in the hypothalamus, exacerbated hypothalamic inflammation. Since ARC neurons in the hypothalamus are directly or indirectly responsible for food intake and metabolism [21], PS-MP-induced hypothalamic inflammation damages ARC neurons, leading to impaired perception of peripheral metabolic signals. This may exacerbate HFD-induced obesity through increased food intake and decreased energy expenditure. 

In addition, neuroinflammation correlates with neurodegeneration and cognitive dysfunction in terms of attention, learning, and memory [22]. It is important to note that fPS-MPs were present in almost all regions of the brain. fPS-MPs were present substantially more in the striatum and hippocampus; the striatum is responsible for movement and Parkinson’s disease, and the hippocampus plays a significant role in learning and memory [23]. Thus, further research investigating the effects of microplastics on Parkinson’s disease, memory, and cognition would be meaningful.

Recently, it has been reported that nanometer-sized particles (approximately 5 nm), which are 1/200 smaller than the particles we used, reach the brain in just 2 h after administration to normal mice [24]. These nanoparticles were rapidly taken up and disappeared after 4 h. They also showed that cholesterol molecules enhanced the uptake of these particles into the membrane of the BBB. Our results suggest that microparticles with an average size of 1.0 µm can also penetrate the BBB, which is fragile under high-fat or high-cholesterol diet conditions, and accumulate in the whole brain region. Once in the brain, these microparticles further exacerbate HFD-induced neuroinflammation. Further research is needed to fully understand the toxicological molecular mechanisms of PS-MP exposure. For example, the most common polymer types of “real-world” microplastics are polyethylene, polypropylene, and polystyrene [7], and this study analyzed only polystyrene, excluding polyethylene and polypropylene, based on results from in silico experimentation. Although in vitro and in vivo experiments with polystyrene showed the significant health effects of polystyrene, additional in vitro and in vivo studies are needed to confirm the effects of polyethylene and polypropylene.

## 4. Materials and Methods

### 4.1. In Silico Experiment

We investigated the binding affinity and binding site between three types of plastic polymers and three major immune cells using in silico molecular docking simulation to investigate which types of MPs polymers can bind to immune cells. For molecular docking analysis, the 3D structures of ethylene (CID 174), propylene (CID 8252), and styrene (CID 7501) were collected from PubChem (https://pubchem.ncbi.nlm.nih.gov/) (Figure 1A–C). Then, 3D crystal structures of representative surface proteins of three target immune cells—the migration inhibitory factor (MIF, PDB ID: 1GD0) for macrophages, T-cell surface glycoprotein CD4 (PDB ID: 1WIP) CD4 T cells, and T cell coreceptor CD8 (PDB ID: 1AKJ) CD8 T cells—were obtained from the PDB database (https://www.rcsb.org).

Molecular docking simulation used AutoDock Vina open-source software to calculate the binding site and binding affinity scores (kcal/mol). A lower binding affinity score implied better binding of MP polymers and immune cells [25]. We then visualized the results of binding structure, site, and affinity using PyMOL [26].

### 4.2. In Vitro Experiment

BV2 murine microglial cells (5 × 10^4^ cells/well) in 96-well plates were cultured in DMEM media and incubated with various concentrations of fluorescence-labeled PS-MPs (fPS-MPs) for 24 h. The fPS-MPs used were latex beads, carboxylate-modified polystyrene, yellow-green fluorescent, 1.0 µm mean particle size, with excitation and emission wavelengths of ~470 and ~505 nm (L4655, Sigma, St. Louis, MO, USA). These fPS-MPs contain 4.75 × 10^10^ beads/mL. Thus, a 0.1% concentration of fPS-MPs contains 4,750,000 MP beads, and a 0.05% concentration contains 2,375,000 MP beads. After 24 h of incubation, the cells were washed with phosphate-buffered saline (PBS, Gibco, Waltham, MA, USA) and stained with 0.5 µg/mL of Hoechst staining solution for 10 min at room temperature. Fluorescent intensities were measured using a microplate reader fluorometer (Gemini XPS/EM, Molecular Device, San Jose, CA, USA). The mean fluorescence index was calculated by dividing the fluorescent intensity at 470 nm/505 nm for fPS-MP by the Hoechst intensity at 355 nm/460 nm.

For fluorescence imaging, the fPS-MP-treated BV2 cells were fixed in 4% paraformaldehyde on a cover slip in 24-well plates. After washing with PBS, the coverslips were mounted with Antifade mounting medium with DAPI (Vectashield, Burlingame, CA, USA), and fluorescence microscopy images were taken with an iRiS Digital Cell Imaging System (Logos Biosystems, Annandale, VA, USA) or a confocal fluorescence microscope (Carl Zeiss, Göttingen, Germany).

### 4.3. In Vivo Experiment

#### 4.3.1. Experimental Design

For in vivo experiments, mice were fed with a normal chow diet (NC, Research Diets Inc., New Brunswick, NJ, USA, Rodent diet with 10 kcal% fat) or a high-fat diet (HFD, Research Diets Inc, Rodent diet with 60% kcal% fat) for four weeks to induce obesity. We then allocated the mice to three groups: the NC group (*n* = 5), the HFD group (*n* = 5), and the MPs groups (HFD plus fPS-MPs 0.125 µg/mouse (2.375 × 10^6^ MPs/mouse), *n* = 5). The 1.0 µm fPS-MPs were orally given daily for six weeks, while the NC and HFD groups were given distilled water (Figure 3A for the experimental scheme). Animal maintenance and MP administration were followed according to the Guide for the Care and Use of Laboratory Animals of NIH, and the Animal Research Ethics Committee approved this study (KHSASP-20-163).

#### 4.3.2. Metabolic Profile Measurements

Each mouse’s body weight was measured using a scale (CAS 2.5D, Seoul, Republic of Korea) at the beginning and end of the experiment. The food intake was calculated using the daily feed intake per mouse by subtracting the remaining feed weight per cage. Calorie intake was calculated by multiplying food intake by 2.91 kcal/g in the NC group and by 5.24 kcal/g in the HFD and PS-MPs groups.

The oral glucose tolerance test (OGTT) and homeostatic model assessment for insulin resistance (HOMA-IR) were measured after overnight fasting at 9 weeks. For OGTT, glucose (2 g/kg body weight) dissolved in distilled water was orally given to each mouse. The blood samples withdrawn from the tail vein were taken at 0, 30, 60, 90, 120, and 180 min after the oral glucose loading. The glucose level was measured using a strip-operated blood glucose meter (ACCU-CHEK Performa, Seoul, Republic of Korea). Before OGTT, blood fasting insulin levels were analyzed using an ultra-sensitive mouse insulin ELISA kit (Crystal Chem, Elk Grove Village, IL, USA). HOMA-IR was determined according to the equation: HOMA-IR = (fasting glucose (mg/dL) × fasting blood insulin (ng/mL))/22.5 [27].

#### 4.3.3. Measurements of Biochemical Parameters

Each mouse was anesthetized, and blood was collected from the heart at week 10. Lipid profiles, including total cholesterol (Total), high-density lipoprotein cholesterol (HDL), and low-density lipoprotein cholesterol (LDL), were measured. Aspartate aminotransferase (AST), alanine aminotransferase (ALT) for hepatic liver function, and creatinine for renal function were measured.

#### 4.3.4. Flow Cytometric Analysis of Blood Immune Cells and Adipose Tissue Macrophages (ATMs)

Blood samples were withdrawn from the tail veins of mice at 9 weeks. After adding EDTA to the blood, 1% FcBlock (BD Biosciences, San Jose, CA, USA) was added to each sample, which was then incubated with fluorophore-conjugated antibodies for 20 min. Antibodies used for blood lymphocyte and Ly6C monocyte analyses were CD45-APC Cyanine7, CD11b-phycoerythrin Cyanine7, CD4-PerCp CY5.5, CD8-phycoerythrin, and Ly6C-APC.

For staining for ATMs, the stromal vascular cells (SVCs) were prepared from the epididymal fat pad at 10 weeks. The fat pad samples were mixed with a solution composed of 2% bovine serum albumin (BSA, Gibco, Waltham, MA, USA) in PBS. The fat pads were minced using a round-shaped scissor into small 1~2 mm pieces. Then, 10 mg/mL of type 2 collagenase (Worthington, Lakewood, NJ, USA) and 2 mg/mL of deoxyribonuclease I (Roche, Indianapolis, IN, USA) were added to the samples and incubated at 37 °C for 30 min with shaking [28]. Then, 5 mM EDTA/2% BSA/PBS solution was added to each tube, filtered to remove undigested adipose pieces with a 100 µm nylon filter (BD Biosciences, San Jose, CA, USA), and centrifuged at 1000 rpm for 3 min. The SVC pellets containing ATMs were suspended with 2% FBS/PBS for staining.

Isolated SVCs were added with 1% Fc Block and then incubated with CD45-APC Cyanine7, CD11b-phycoerythrin Cyanine7, and F4/80-APC, CD11c-phycoerythrin. All antibodies were purchased from BioLegend (San Diego, CA, USA). After washing with a 2% FBS/PBS solution and centrifuging at 1500 rpm, we analyzed them using flow cytometry with BD Canto (BD Biosciences, San Jose, CA, USA).

The proportions of immune cells were analyzed using the FlowJo software (Tree Star, Inc., Ashland, OR, USA). We identified CD45^+^, CD11b^+^, and F4/80^+^ for ATMs; CD45^+^, CD11b^+^, F4/80^+^, and CD11c^+^ for M1 ATMs; CD45^+^ and CD4^+^ for CD4 T lymphocytes; CD45^+^ and CD8^+^ for CD8 T cells; CD45^+^ and CD11b^+^ for monocytes; CD45^+^, CD11b^+^, and Ly6C high levels for Ly6C^high^ monocytes; CD45^+^, CD11b^+^, and Ly6C low levels for Ly6C^low^ monocytes. fPS-MPs were detected in the FITC spectrum.

#### 4.3.5. Fat Accumulation Analysis of the Liver and Epididymal Fat Pad

Epididymal fat pads and liver were fixed in 4% paraformaldehyde (PFA) and prepared into paraffin-embedded blocks. We cut each paraffin block into 5 μm thick slices and put each on a microscope slide. For hematoxylin and eosin (H&E) staining, two slides per mouse were deparaffinized and hydrated by a gradient. The images of the H&E stain were taken with a BX50 microscope (Olympus Optical, Tokyo, Japan). Then, we calculated the adipocyte size of the epididymal fat pad and the % of the area of lipid droplets in the liver with ImageJ.

#### 4.3.6. Brain Tissue Immunohistochemistry Staining

Whole brains were separated from the skull, fixed overnight with PFA, and then placed in a 30% sucrose/0.05 M PBS solution at 4 °C. The brains were cryo-sectioned using a Cryostat (Microsystems AG, Leica, Wetzlar, Germany) with 30 μm thick coronal sections and stored in a cryoprotectant solution consisting of 0.2 M PB, 25% ethylene glycol, 25% glycerol, and water at 4 °C [29]. All sections were collected in six different series and processed for immunostaining as previously described [30].

Brain coronal cryosections (30 μm in thickness) containing the hypothalamus were incubated with rabbit anti-glial fibrillary acidic protein antibody (anti-GFAP, 1:5000; Neuromics, Edina, MN, USA) for astrocytes or rabbit anti-ionized calcium-binding adaptor molecule 1 antibody (anti-Iba-1, 1:1000; Wako, Osaka, Japan) for microglia [31]. Then, it was stained with biotinylated goat anti-rabbit IgG secondary antibody and detected with the avidin-biotin complex (ABC) standard kit (Vector Laboratories, Burlingame, CA, USA). Then, 0.5 mg/mL 3,3′-diaminobenzidine (Sigma, St. Louis, MO, USA) in 0.003% H_2_O_2_/0.1 M PBS was added to sections to visualize the antibody-positive signals. To quantify GFAP- or Iba-1-positive cells, the images of stained brain sections were taken by a microscope (Olympus Optical, Tokyo, Japan).

#### 4.3.7. Analysis of Activated Astrocytes and Microglia

To quantify resting and activated microglia and astrocytes in the hypothalamus, coronal brain sections (30 μm thick) were collected (5 sections/series), labeled with anti-Iba-1 or anti-GFAP antibodies, and imaged under a bright-field microscope (Olympus Optical, Tokyo, Japan). The coronal brain sections labeled with anti-GFAP or anti-Iba-1 were digitized and manually counted within preselected fields (500 × 400 μm) of the hypothalamus (2 fields/animal). Activated microglia and astrocytes were classified and counted according to their morphologies, as described [15]. In detail, resting microglia and astrocytes exhibited small-shaped soma that exhibited long, thin, and ramified processes. In contrast, activated microglia and astrocytes exhibited enlarged-swollen cell soma and retracted processes, such that the length of the process was less than the diameter of the cell soma. Only cells with clear nuclei and clear boundaries were selected for analysis [32].

#### 4.3.8. Detection of fPS-MPs in the Blood and Brain

The fPS-MPs used in the in vivo experiment were green fluorescent beads with excitation and emission wavelengths of ~470 and ~505 nm, respectively, which is close to FITC’s absorbance of ~495 nm and emission of ~525 nm. Thus, fPS-MPs can be excited by a blue laser and emit green fluorescence light.

Blood samples were stained with fluorophore antibodies except for FITC, washed with 2% FBS/PBS solution, and then analyzed using flow cytometry with BD Canto (BD Biosciences, Franklin Lakes, NJ, USA). fPS-MPs in the blood were detected in the FITC spectrum of flow cytometric analysis.

Brain coronal cryosections were mounted with GEL/MOUNT with DAPI (Biomed, Foster City, CA, USA), and the images were captured with the iRiS Digital Cell Imaging System. fPS-MPs deposited in the brain were detected in the FITC spectrum of fluorescence microscopic analysis.

#### 4.3.9. Statistical Analysis

All experimental data are presented as means ± standard error of the mean (SEM). All statistical analyses and graph creations were accomplished by GraphPad Prism 5 (GraphPad Software, San Diego, CA, USA). Tukey’s post hoc test with one-way analysis of variance (ANOVA) was used to explore differences between groups. All *p*-values were two-tailed, and *p* < 0.05 was considered statistically significant.

## 5. Conclusions

Among the three types of microplastic polymers, polystyrene is most likely to bind to immune cells in silico, and 1 μm sized fluorescently labeled polystyrene microplastic (fPS-MP) particles were indeed phagocytized by BV2 microglial cells. In HFD-induced obese mice, oral administration of fPS-MPs aggravated abnormal fat accumulation, impaired glucose metabolism, and insulin resistance. fPS-MPs also increased Ly6C^high^ inflammatory monocytes in the blood and inflammatory CD11c^+^ macrophages in the adipose tissue. fPS-MP particles were detected throughout the brain and increased the number of activated microglia in the hypothalamus. We conclude that fPS-MPs contribute to the exacerbation of glucose metabolism and insulin resistance by increasing obesity-related peripheral and central inflammation. This highlights the potential for fPS-MPs exposure to significantly impact obesity and brain health in humans.
